# Pharmacological therapy for central giant cell granuloma of the jaws: A systematic review

**DOI:** 10.4317/jced.61490

**Published:** 2024-07-01

**Authors:** Fernando-Aguiar Corrêa, José-Alcides-Almeida de Arruda, Victor-Zanetti Drumond, Isadora-Vilas-Boas Cepeda, Sandra-Beatriz-Chaves Tarquinio, Tarcília-Aparecida Silva, Lucas-Guimarães Abreu, Elena-Riet-Correa Rivero, Ricardo-Alves Mesquita, Adriana Etges

**Affiliations:** 1 Postgraduate Program in Dentistry, Universidade Federal de Pelotas, Pelotas, Brazil; 2 Department of Oral Diagnosis and Pathology, School of Dentistry, Universidade Federal do Rio de Janeiro, Rio de Janeiro, Rio de Janeiro, Brazil; 3 Private Practice Dentistry, Ipatinga, Minas Gerais, Brazil; 4 Diagnostic Center for Oral Diseases, School of Dentistry, Universidade Federal de Pelotas, Pelotas, Brazil; 5 Department of Oral Surgery, Pathology, and Clinical Dentistry, School of Dentistry, Universidade Federal de Minas Gerais, Belo Horizonte, Brazil; 6 Department of Child and Adolescent Oral Health, School of Dentistry, Universidade Federal de Minas Gerais, Belo Horizonte, Brazil; 7 Department of Pathology, Health Sciences Center, Universidade Federal de Santa Catarina, Florianópolis, Brazil

## Abstract

**Background:**

Pharmacological therapy has been used as an alternative or complementary approach to surgery in central giant cell granuloma (CGCG) of the jaws. This systematic review examined the effectiveness of pharmacological therapy for CGCG of the jaws, focusing on clinical outcomes.

**Material and Methods:**

Electronic searches were performed in six databases. Case reports and/or cases series were included. The Kaplan-Meier survival analysis method was used to evaluate outcomes related to clinical resolution and recurrence. The risk of bias was assessed using the Joanna Briggs Institute tool.

**Results:**

A total of 74 studies comprising 205 cases of CGCG were included. About 65.4% of cases occurred in individuals under 20 years of age. Most of the treated patients were women (61%) and the mandible (72.2%) was the most reported site. Curettage and enucleation before or after pharmacological therapy were reported in 28.3% and 19% of cases, respectively. The main pharmacological agent used was triamcinolone (37.5%). Complete resolution of CGCG was reported at a rate of 77.1%, while side effects were experienced by 9.8% of individuals. The recurrence rate was 6.8%.

**Conclusions:**

Pharmacological therapy may be an effective and safe option for managing CGCG, especially in the young population. Although the overall success rate in achieving complete resolution is encouraging, further controlled studies are needed to refine drug selection and protocols.

** Key words:**Calcitonin, Central giant cell lesion, Denosumab, Interferon, Pharmacological therapy, Triamcinolone.

## Introduction

Central giant cell granuloma (CGCG) is a localized and benign but occasionally aggressive osteolytic lesion of the jaws (1,2). First described by Jaffe in 1953 (3), this lesion remains uncertain in terms of origin and etiology. Factors such as a reactive origin to a local irritant, developmental anomaly, and neoplasia have been proposed (4,5). Histopathologically, CGCG is characterized by the proliferation of osteoclast-like giant cells and a mononuclear cell population composed of macrophage/monocytic cells and spindle-shaped mesenchymal cells (2,6). CGCG has a striking predilection for females and the mandible, and about 50% of affected subjects are children and adolescents (7).

CGCG are usually painless, slow-growing lesions that do not affect vital structures, i.e., so-called non-aggressive lesions (7). Aggressive CGCG is characterized by pain, rapid growth, root resorption, cortical perforation, with an average diameter of 5.8 cm, and a high recurrence rate (1). Recent literature has reported challenges in treating aggressive lesions compared to non-aggressive ones (5,7). Conservative surgical treatment (e.g., enucleation and curettage) has been the mainstay approach to CGCG (7). Nevertheless, its limitations concern the significant probability of recurrence and esthetic and functional impairment, particularly in children, regarding maxillofacial development and growth (8,9).

Pharmacological therapy has been used as an alternative or complementary approach to surgery in CGCG, with the purpose of promoting healing and reducing size, thus minimizing damage caused by extensive surgical procedures and the risk of recurrence (7,10). Different drugs (e.g., triamcinolone, interferon, calcitonin, and denosumab) with multiple protocols have been described elsewhere (5,7,10,11). Two previous systematic review have been published on the topic. The first was in 2009 (12), while a recent analysis synthesized data from 15 studies on non-surgical treatments for CGCG (10). However, isolated case reports were not incorporated in the latter review (10). In particular, it is known that publishing case reports has become increasingly difficult; thus, the motivation to revisit existing literature without restrictions of study designs was based on the premise that unusual diseases treated with alternative approaches along with long-term follow-up, when well documented, can make an important contribution to decision making (14).

The aim of the present systematic review was to evaluate the pharmacological therapy used in CGCG of the jaws. Our focus was to examine information on complete or partial clinical resolution, side effects, recurrence rate, and need for additional interventions.

## Material and Methods

-Eligibility criteria

The answer to the following question: “what are the pharmacological therapies that have been indicated for the treatment of CGCG of the jaws?”, was investigated based on the PICO framework: *P* (population): patients with CGCG of the jaws; I (intervention): pharmacological therapy; C (comparison): not applicable; and O (outcome): total or partial clinical resolution of the lesion, side effects, recurrence, and need for another intervention.

Studies published in English were included. Articles such as case reports or case series with sufficient data about the cytopathological and/or histopathological diagnosis of CGCG of the jaws treated with pharmacological agents were included. Exclusion criteria were articles whose data could not be extracted, experimental studies, letters to the editor, and expert opinions/comments, unless any of these types of articles provided sufficient and detailed data of interest. Disorders related to CGCG of the jaws, such as brown tumors of hyperparathyroidism, cherubism, and syndromes such as Noonan and LEOPARD, or neurofibromatosis type 1 were not considered.

-Search scheme

PubMed, Web of Science, Ovid, Embase, Cochrane Library, and Scopus were consulted without time constraints in July 2022. Search updates were made in October 2023 according to the strategy used in the databases with Boolean operators linking terms and keywords. Adjustments were applied to the search scheme according to the characteristics of each database ( Table 1). Hand searches were also undertaken by cross-checking the reference lists of the included articles. Duplicate references encountered in different databases were removed using the EndNote program (EndNote®, Clarivate Analytics, Toronto, Canada).

-Study selection

The studies were selected by two independent authors (F.A.C. and V.Z.D.) in two phases. In the first phase, the two authors evaluated the studies based on their titles and abstracts, and those that fulfilled the eligibility criteria were included. The full text of the articles without sufficient information in the titles/abstracts was acquired in order to permit the authors to decide whether to include or exclude. In the second phase, the authors evaluated the complete texts and included those that met the eligibility criteria. Disagreements were resolved by a discussion with other authors (E.R.C.R., R.A.M., and A.E.).

-Data collection process and items

Data collection was performed independently by two reviewers and then cross-checked. For each study included, data referring to the surname of the first author, year of publication, study design, country where the study was performed, sample size, age and sex of the participants, anatomical location (maxilla or mandible), time of evolution, symptomatology, lesion size, imaging aspects, previous treatments (e.g., surgical and/or pharmacological interventions), current treatment (i.e., concentration, dose, route of administration, administration interval, time of use), additional treatment, clinical resolution, side effects, recurrence, and follow-up period.

-Classification of CGCG aggressiveness

The cases reported in the included studies were classified according to aggressiveness. For cases in which the authors did not classify the clinical behavior of aggressive and non-aggressive lesions, the criteria adopted by Chuong *et al*. (1) and Kaban *et al*. (14) were followed.

-Risk of bias assessment

The Joanna Briggs Institute (University of Adelaide) tools for case reports or case series were employed (15). The articles were appraised by two authors (F.A.C. and A.E.) according to the following parameters: demographic data of the patient’s characteristics, medical history and presentation as a timeline, current clinical condition of the patient, diagnostic tests and evaluation method, treatment provided, information about the post-intervention clinical picture, and identification or list of side effects. For each parameter, the risk of bias of each included article was defined as “yes” (low), “no” (high), or “not applicable”.

-Data analysis

Data were tabulated in Microsoft Office Excel 2019 (Microsoft® software, Redmond, WA, USA) and analyzed descriptively. The MedCalc software (MedCalc software bvba, Ostend, Flander, Belgium) was used to construct the Kaplan-Meier survival curves regarding total clinical resolution and recurrences.

-Protocol and registration

This systematic review was performed according to the Preferred Reporting Items for Systematic Reviews and Meta-analyses (PRISMA) guidelines (16). A protocol was drafted and registered with the National Institute for Health Research International Prospective Register of Systematic Reviews (PROSPERO; https://www.crd.york.ac.uk/prospero/) under registration number CRD42021266588.

## Results

-Study selection

**Figure 1:**
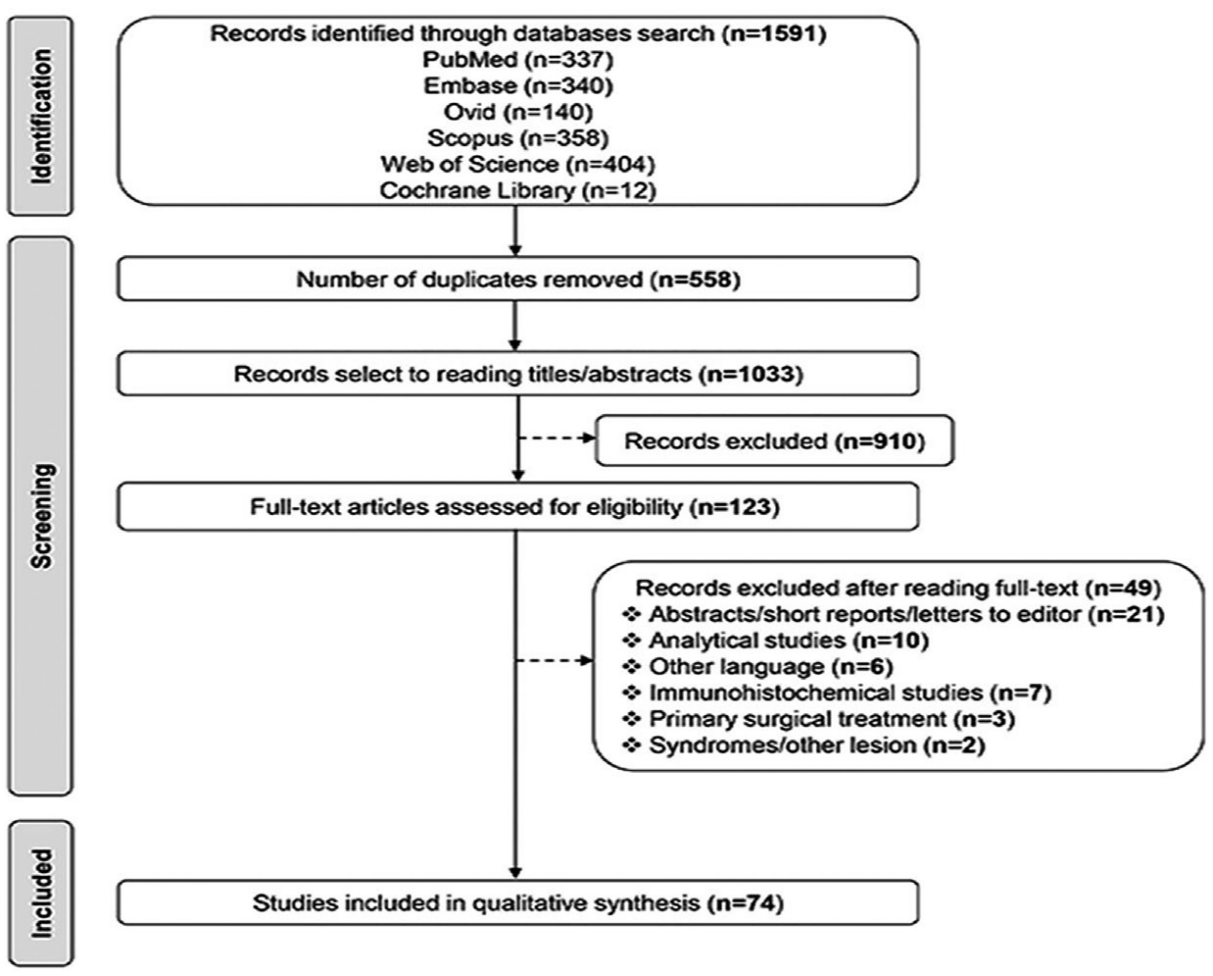
Flowchart showcasing the screening procedure.

The electronic searches yielded 1591 articles. After removing duplicates, 1033 articles remained. After a comprehensive evaluation of titles and abstracts, 143 studies were eligible, 69 of which were excluded after reading the full text. Subsequently, 74 studies with a total sample of 205 cases of CGCG of the jaws were included for qualitative analysis. A flowchart depicting the search of articles and the selection process is shown in Figure 1.

-Study characteristics

**Figure 2:**
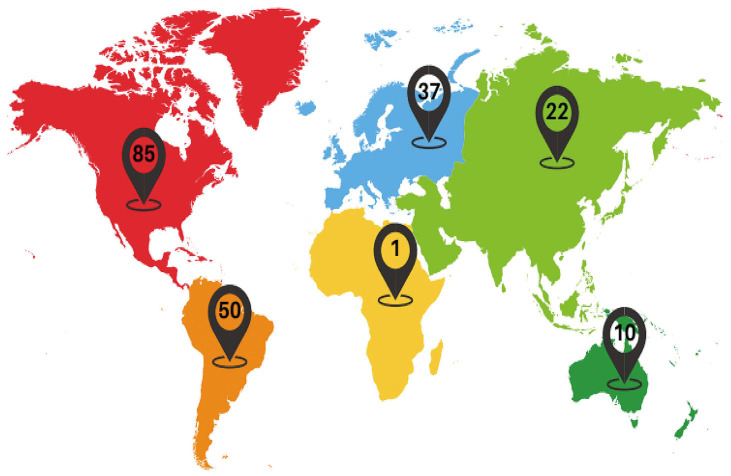
Global distribution of central giant cell granulomas of the jaws managed with pharmacological therapy.

Of the 74 studies included, 57 were case reports (17-73) and 17 were case series (74-90). Case reports were published between 1998 and 2023, while case series were published between 1993 and 2023. The smallest case series comprised three individuals and the largest 45 individuals. North America was the continent with the highest number of cases published in the literature (Fig. 2).

-Risk of bias of studies

**Figure 3:**
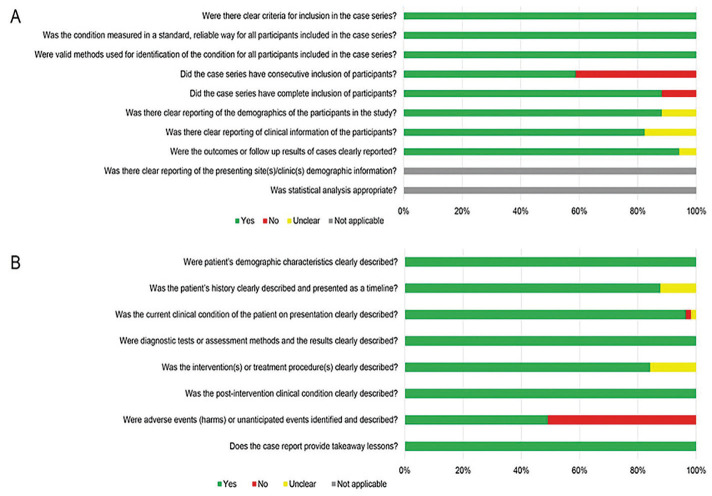
Graphical illustration of the risk of bias appraisal.

The studies were classified as having low or high risk of bias. A high risk of bias was detected in one of the 17 case series ( Table 2) and in 11 of the 57 case reports ( Table 3). Figure 3 showcases a graphical representation of the results from the risk of bias assessment.

-Clinicodemographic data

The mean age of individuals affected by CGCG was 18.1 ± 12.3 years. Women (61.0%) were more affected than men (39.0%). Most cases occurred in the mandible (72.2%) and in the anterior portion (44.4%), with a 59.5% prevalence of lesions with aggressive behavior. Pain and swelling were reported in 7.8% and 36.1% of cases, respectively. Detailed information regarding clinicodemographic data is provided in  Table 4.

-Treatment and pharmacological approach

Surgical treatments prior to current pharmacological therapy were performed in 29.8% of cases, with conservative surgery (curettage and/or enucleation) being the most common (28.3%). Pharmacological therapy was previously used in the current treatment in 3.9% of cases. Drug combinations were administered to 15 (7.3%) individuals. The most common route of administration was subcutaneous (45.4%), followed by the intralesional one (39.0%).

Triamcinolone (concentration range: 10–40 mg) was used exclusively in 77 (37.5%) cases. The total dose range administered was 40–3120 mg. The time of use varied from 0.03 to 20 months. Interferon (15 mcg concentration and 9 MIU/m²) was used exclusively in 50 (24.4%) cases. The total dose range administered was 7020 mcg and 1095 MIU/m². The time of use varied from one to 14 months. Calcitonin (concentration range: 50–200 IU) was used exclusively in 30 (14.7%) cases. The total dose range administered was 74200–602000 IU. The time of use varied from 1.3 to 60 months. Denosumab (concentration range: 60–120 mg) was used exclusively in 25 (12.2%) cases. The total dose range administered was 60–1800 mg. The time of use varied from 0.03 to 12 months. Other drugs such as prednisolone, zoledronic acid, hydrocortisone, alendronate, flucortolone, and solumedrol were also reported.

Drug combinations were reported in 15/205 (7.3%) cases, in which at least two drugs were administered. Of these, 7/29 (24.1%) cases were triamcinolone with denosumab, 4/29 (13.79%) were triamcinolone with calcitonin, and 4/29 (13.79%) were zoledronic acid with interferon. Of the current treatments using drug associations, 8/15 (53.3%) were for aggressive lesions. Adverse effects were reported in 20/205 (9.8%) individuals, the most common being hypercalcemia, nausea, and hypocalcemia.

In 46/205 cases (22.4%) additional surgery was performed after current pharmacological therapy. Conservative surgery (curettage and/or enucleation) was performed in 39/205 (19.0%) cases, followed by aggressive surgical procedures (resection) in 7/205 (3.4%) cases.

Immunohistochemical analysis complementary to pharmacological treatment was performed in 5/205 (2.4%) cases. The markers analyzed were calcitonin and glucocorticoid receptors, RANKL, and CD34.

-Outcomes

Regarding the clinical/image resolution outcome, data of 200 individuals with information about outcome and follow-up were available; 158 exhibited total clinical/imaging resolution and 42 exhibited partial clinical/imaging resolution during the follow-up period. Of a total of 47 lesions that had exhibited partial resolution with current pharmacological therapy, 36/47 (76.5%) were aggressive.

**Figure 4:**
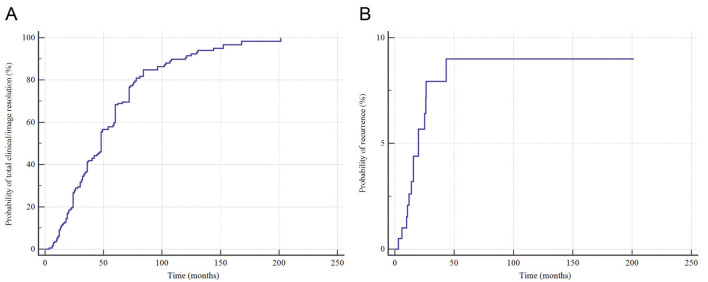
Outcomes of central giant cell granulomas of the jaws managed with pharmacological therapy. (A) Probability of lesion resolution and (B) probability of recurrence in relation to follow-up time.

Mean follow-up time was 56.0 months (standard error = 3.2). The probability of total clinical/imaging resolution at 12 months of follow-up was 9.1%. Within the 144-month follow-up, the probability of total clinical/image resolution increased markedly to 95.0% (Fig. 4A).

Data from 199 individuals were pooled for the recurrence outcome. Mean follow-up time was 185.2 months (standard error = 4.24). The probability of recurrence at six months of follow-up was 1.0%. Within a 26.4-month follow-up, the probability of recurrence was 7.9% (Fig. 4B). Of the 14 cases with recurrence, six (42.9%) were treated only with interferon and 13 (92.9%) were aggressive.

## Discussion

The objective of the present systematic review was to gather relevant data in order to assist clinicians and surgeons in choosing the best protocols for treating patients with CGCG of the jaws. In line with previous literature, CGCG occurred more frequently in young women and the mandible was reported as the most commonly affected site. Pain and swelling were the most frequently reported signs and symptoms associated with this condition (7,10). Most cases of CGCG examined in this systematic review were classified as having an aggressive behavior. This classification suggests that these cases may have been more destructive or fast-growing in nature, with consequent implications for treatment decisions (1,14). When considered aggressive, CGCG requires a more extensive surgical procedure and tends to have a greater chance of recurrence when compared to non-aggressive lesions (22.8% vs. 7.8%, respectively) (9). Multinucleated giant cells are a characteristic feature of CGCG and are believed to play a role in the pathogenesis and aggressiveness of the lesion, particularly with osteoclast-like activity (6). The specific mechanisms by which multinucleated giant cells contribute to CGCG aggressiveness are still unknown. The production of cytokines and growth factors related to inflammation and the recruitment of immune cells have been linked to the aggressive nature of these lesions (91).

Intralesional injections of triamcinolone acetonide were employed at varying concentrations (10, 20 and 40 mg) to manage CGCG cases. The concentration of 40 mg seems to have been the most effective in achieving a total resolution of the condition (92). Triamcinolone acetonide is a synthetic corticosteroid drug with anti-inflammatory and immunosuppressive properties. The impact of triamcinolone acetonide on bone varies depending on dosage, duration of use, and individual susceptibility (56,86). Notably, a recent study found no significant differences in glucocorticoid receptor immunoexpression in mononuclear stromal cells or multinucleated giant cells with respect to the aggressiveness of CGCG or its response to clinical treatment with triamcinolone (93). The advantages of this therapy are its less invasive nature, the likely lower cost for the patient, a lower risk, and the ability to treat the lesion surgically in the future, if necessary (56). Conversely, some authors have emphasized that high or prolonged doses of this corticosteroid have been associated with an increased risk of osteoporosis, fractures, and even avascular necrosis of long bones (5,56,86).

Interferon and calcitonin were introduced as alternative pharmacological therapies for the management of CGCG in the 1990s (11,14,33,80). Interferon therapy has been investigated as a potential inhibitor of giant cell growth and activity in CGCG due to its immunomodulatory effects, particularly in controlling lesion progression (14,23,33,41). Calcitonin is a hormone that regulates calcium and bone metabolism and has been used in the treatment of diseases involving bone resorption, such as osteoporosis (94). In the case of CGCG, calcitonin has been considered a treatment option because it might help inhibit bone resorption, which is a hallmark of CGCG (5). The present systematic review identified that the use of interferon varied greatly in terms of the concentration and total dose applied. Furthermore, most cases received interferon before lesion enucleation. Of the 50 cases exclusively treated with interferon, 8 (16.0%) showed partial resolution and required additional treatment (5,58,70). On the other hand, 83.3% of 30 cases treated with calcitonin exhibited complete resolution of the lesion. In this line, low recurrence rates were reported when compared to curettage (9.1% vs. 53.8%, respectively) in aggressive cases of CGCG (95). However, the disadvantage of calcitonin therapy is that it may have to be administered for more than two years and the route is usually nasal (5,95).

Recently, denosumab was approved for the treatment of common metabolic bone diseases and has been used off-label in rare metabolic bone diseases (96). Denosumab is a monoclonal antibody against the receptor activator of nuclear factor-κB (RANK) ligand (RANKL) which substantially suppresses osteoclast formation and activity (96). So far, about 25 patients with CGCG have been treated with denosumab. Studies have documented a reduction in the size of the lesion, decreased pain, and stabilization of the affected bone (90). The average complete treatment time was 10.5 months, a relatively short period when compared to other therapies that used just one drug. However, it is important to highlight that the choice of denosumab for CGCG depends particularly on the location and aggressiveness of the lesion, in addition to the healthcare provider’s experience (96). This is because denosumab-related adverse effects such as osteonecrosis, hypercalcemia, and increased serum urea and creatinine levels have been reported elsewhere. Another important factor to consider is the high cost of this drug (90,96).

The use of pharmacological therapy for the management of CGCG is a valuable strategy, both as an adjunct to surgical treatment and as the primary treatment in certain cases (5,7). The data provided indicated that overall recurrence after treatment occurred in almost 7% of cases. Recurrence is a significant concern in CGCG (9), and this relatively low recurrence rate suggests that a combination of surgical and pharmacological approaches has been effective in managing the condition. Nonetheless, a significant proportion of individuals (i.e., 53.3%) treated with drug association had lesions classified as aggressive. This suggests that, in aggressive cases, the combination of pharmacological therapy with surgery may be a preferred approach addressing the complexity of the condition and reducing the risk of recurrence. It is also necessary to consider possible side effects (e.g., hypercalcemia, hypocalcemia, nausea, and osteonecrosis), patient age, and treatment costs. Additionally, ossification caused by the use of corticosteroids, especially triamcinolone, can also be considered a side effect, potentially leading to the need for subsequent osteoplasty (56,86).

Since most of the studies available in the literature were cases reports, certain limitations and heterogeneity of the present review should be acknowledged such as the fact that different dosages and concentrations of medication were employed and that detailed information about the treatment period, image characteristics at follow-up, and the measurement of the size of the lesion were not standardized.

In summary, pharmacological therapy is considered to be a viable treatment option for CGCG of the jaws, with a relatively high rate of complete resolution. However, healthcare providers should closely monitor patients during treatment to ensure the best outcomes and to minimize any potential adverse effects. Further controlled studies are needed to refine drug selection and protocols considering the aggressiveness of the lesion.

## Figures and Tables

**Table 1:** Search strategy employed to identify articles in electronic databases.

PubMed and Web of Science	giant cell granuloma OR Central giant cell granuloma OR giant cell reparative granuloma OR central giant cell lesion OR CGCL OR CGCG AND calcitonin OR denosumab OR Triamcinolone acetonide OR triamcinolone hexacetonide OR Prolia OR "Xgeva" OR AMG 162 OR "Calcitrin" OR Thyrocalcitonin OR Ciba OR "Cinonide" OR "Tricort" OR Azmacort OR Kenacort OR Kenalog OR "Volon" OR Interferon OR Pegasys OR Imatinib mesylate OR Dasatinib OR nilotinib OR Bosutinib OR glivec OR gleevec OR STI571 OR CGP 57148
Embase and Scopus	"giant cell granuloma" OR "central giant cell granuloma" OR "giant cell reparative granuloma" OR "central giant cell lesion" OR CGCL OR CGCG AND calcitonin OR denosumab OR ‘triamcinolone acetonide" OR "triamcinolone hexacetonide" OR prolia OR xgeva OR "AMG 162" OR calcitrin OR thyrocalcitonin OR ciba OR cinonide OR tricort OR azmacort OR kenacort OR kenalog OR volon OR interferon OR pegasys OR "imatinib mesylate" OR dasatinib OR nilotinib OR bosutinib OR glivec OR gleevec OR STI571 OR "CGP 57148"
Cochrane Library and Ovid	giant cell granuloma OR central giant cell granuloma OR giant cell reparative granuloma OR central giant cell lesion OR CGCL OR CGCG AND calcitonin OR denosumab OR triamcinolone acetonide OR triamcinolone hexacetonide OR prolia OR xgeva OR AMG 162 OR calcitrin OR thyrocalcitonin OR ciba OR cinonide OR tricort OR azmacort OR kenacort OR kenalog OR volon OR interferon OR pegasys OR imatinib mesylate OR dasatinib OR nilotinib OR bosutinib OR glivec OR gleevec OR STI571 OR CGP 57148

**Table 2:** Joanna Briggs Institute (JBI) critical appraisal checklist for case series.

Study	Items										Risk of bias
	Q1	Q2	Q3	Q4	Q5	Q6	Q7	Q8	Q9	Q10	
Allon et al. (74)	Yes	Yes	Yes	Yes	Yes	Yes	Yes	Yes	NA	NA	Low
Borges et al. (75)	Yes	Yes	Yes	No	Yes	Yes	Yes	Yes	NA	NA	Low
Bredell et al. (76)	Yes	Yes	Yes	No	Yes	Yes	Yes	Yes	NA	NA	Low
Carlos & Sedano (77)	Yes	Yes	Yes	Yes	Yes	Yes	Yes	Yes	NA	NA	Low
Chandna et al. (78)	Yes	Yes	Yes	Yes	Yes	Yes	Yes	Yes	NA	NA	Low
Choe et al. (79)	Yes	Yes	Yes	Yes	Yes	Yes	Yes	Yes	NA	NA	Low
de Lange et al. (80)	Yes	Yes	Yes	No	Yes	Yes	Yes	Yes	NA	NA	Low
Dolanmaz et al. (81)	Yes	Yes	Yes	No	Yes	Yes	Yes	Yes	NA	NA	Low
Harris (82)	Yes	Yes	Yes	No	No	Yes	Yes	Yes	NA	NA	High
Kim et al. (83)	Yes	Yes	Yes	No	Yes	Yes	Yes	Yes	NA	NA	Low
Nogueira et al. (56)	Yes	Yes	Yes	Yes	Yes	Yes	Unclear	Yes	NA	NA	Low
Niedzielska et al. (85)	Yes	Yes	Yes	Yes	Yes	Yes	Yes	Yes	NA	NA	Low
Nogueira et al. (86)	Yes	Yes	Yes	Yes	Yes	Yes	Unclear	Yes	NA	NA	Low
Pogrel (87)	Yes	Yes	Yes	No	Yes	Yes	Yes	Yes	NA	NA	Low
Rhou et al. (88)	Yes	Yes	Yes	Yes	No	Unclear	Yes	Unclear	NA	NA	High
Schreuder et al. (89)	Yes	Yes	Yes	Yes	Yes	Yes	Yes	Yes	NA	Yes	Low
Vanderniet et al. (90)	Yes	Yes	Yes	Yes	Yes	Unclear	Unclear	Yes	NA	Yes	Low

Note: NA, not applicable. Q1: Were there clear criteria for inclusion in the case series? Q2: Was the condition measured in a standard, reliable way for all participants included in the case series? Q3: Were valid methods used for identification of the condition for all participants included in the case series? Q4: Did the case series have consecutive inclusion of participants? Q5: Did the case series have complete inclusion of participants? Q6: Was there clear reporting of the demographics of the participants in the study? Q7: Was there clear reporting of clinical information of the participants? Q8: Were the outcomes or follow up results of cases clearly reported? Q9: Was there clear reporting of the presenting site(s)/clinic(s) demographic information? Q10: Was statistical analysis appropriate?

**Table 3:** Joanna Briggs Institute (JBI) critical appraisal checklist for case report.

Study	Items								Risk of Bias
	Q1	Q2	Q3	Q4	Q5	Q6	Q7	Q8	
Adornato et al. (17)	Yes	Yes	Yes	Yes	Yes	Yes	No	Yes	Low
Al-Ahmad et al. (18)	Yes	Yes	Yes	Yes	Unclear	Yes	No	Yes	High
Al-Jandan (19)	Yes	Yes	Yes	Yes	Yes	Yes	No	Yes	Low
Al-Layla & Mahazta (20)	Yes	Yes	Yes	Yes	Unclear	Yes	No	Yes	High
Aoki et al. (21)	Yes	Yes	Yes	Yes	Yes	Yes	No	Yes	Low
Aurora et al. (22)	Yes	Yes	Yes	Yes	Yes	Yes	Yes	Yes	Low
Baker et al. (23)	Yes	Yes	Yes	Yes	Unclear	Yes	No	Yes	High
Bayar & Ak (24)	Yes	Yes	Yes	Yes	Yes	Yes	Yes	Yes	Low
Cavalcante et al. (25)	Yes	Unclear	Yes	Yes	Yes	Yes	No	Yes	High
Chien et al. (26)	Yes	Unclear	Yes	Yes	Unclear	Yes	Yes	Yes	Low
Choi & Kraut (27)	Yes	Yes	Yes	Yes	Yes	Yes	No	Yes	Low
Comert et al. (28)	Yes	Yes	Yes	Yes	Yes	Yes	Yes	Yes	Low
da Rosa et al. (29)	Yes	Yes	Yes	Yes	Yes	Yes	No	Yes	Low
da Silva et al. (30)	Yes	Unclear	Yes	Yes	Yes	Yes	No	Yes	High
da Silva Sampieri et al. (31)	Yes	Unclear	Yes	Yes	Yes	Yes	Yes	Yes	Low
de Arruda et al. (32)	Yes	Yes	Yes	Yes	Yes	Yes	Yes	Yes	Low
de Lange et al. (33)	Yes	Yes	Yes	Yes	Unclear	Yes	No	Yes	High
de Mendonça et al. (34)	Yes	Yes	Yes	Yes	Yes	Yes	Yes	Yes	Low
de Oliveira et al. (35)	Yes	Yes	Yes	Yes	Yes	Yes	No	Yes	Low
de Oliveira et al. (36)	Yes	Yes	Yes	Yes	Yes	Yes	No	Yes	Low
El Hadidi et al. (37)	Yes	Yes	Yes	Yes	Unclear	Yes	Yes	Yes	Low
Fernandes Gonçalves et al. (38)	Yes	Yes	Yes	Yes	Yes	Yes	No	Yes	Low
Ferretti & Muthray (39)	Yes	Yes	Yes	Yes	Yes	Yes	No	Yes	Low
Fonseca et al. (40)	Yes	Unclear	Yes	Yes	Yes	Yes	No	Yes	High
Goldman et al. (41)	Yes	Yes	Yes	Yes	Yes	Yes	Yes	Yes	Low
Goyal et al. (42)	Yes	Yes	Yes	Yes	Yes	Yes	No	Yes	Low
Gupta et al. (43)	Yes	Yes	Yes	Yes	Yes	Yes	Yes	Yes	Low
Jerkins et al. (44)	Yes	Yes	Yes	Yes	Unclear	Yes	Yes	Yes	Low
Joshi et al. (45)	Yes	Yes	Yes	Yes	Yes	Yes	Yes	Yes	Low
Khafif et al. (46)	Yes	Yes	Yes	Yes	Yes	Yes	No	Yes	Low
Kurtz et al. (47)	Yes	Unclear	Yes	Yes	Yes	Yes	No	Yes	High
Lietman & Levine (48)	Yes	Yes	No	Yes	Unclear	Yes	No	Yes	High
Mariz et al. (49)	Yes	Unclear	Yes	Yes	Yes	Yes	Yes	Yes	Low
Matos et al. (50)	Yes	Yes	Yes	Yes	Yes	Yes	No	Yes	Low
Mohanty et al. (51)	Yes	Yes	Yes	Yes	Yes	Yes	No	Yes	Low
Moura et al. (52)	Yes	Yes	Yes	Yes	Unclear	Yes	Yes	Yes	Low
Mukdad et al. (53)	Yes	Yes	Yes	Yes	Yes	Yes	Yes	Yes	Low
Naidu et al. (54)	Yes	Yes	Yes	Yes	Yes	Yes	Yes	Yes	Low
Nilesh et al. (55)	Yes	Yes	Yes	Yes	Yes	Yes	No	Yes	Low
Maia Nogueira et al. (84)	Yes	Yes	Yes	Yes	Yes	Yes	No	Yes	Low
O'Connell et al. (58)	Yes	Yes	Yes	Yes	Yes	Yes	Yes	Yes	Low
O'Connell et al. (57)	Yes	Yes	Yes	Yes	Yes	Yes	Yes	Yes	Low
O'Regan et al. (59)	Yes	Yes	Yes	Yes	Yes	Yes	Yes	Yes	Low
Pogrel et al. (60)	Yes	Yes	Yes	Yes	Yes	Yes	No	Yes	Low
Rachmiel et al. (61)	Yes	Yes	Yes	Yes	Yes	Yes	No	Yes	Low
Rajeevan et al. (62)	Yes	Yes	Yes	Yes	Yes	Yes	No	Yes	Low
Romero et al. (63)	Yes	Yes	Yes	Yes	Yes	Yes	Yes	Yes	Low
Schreuder et al. (64)	Yes	Yes	Yes	Yes	Unclear	Yes	Yes	Yes	Low
Schütz et al. (65)	Yes	Yes	Yes	Yes	Yes	Yes	Yes	Yes	Low
Sezer et al. (66)	Yes	Yes	Yes	Yes	Yes	Yes	Yes	Yes	Low
Shirani et al. (67)	Yes	Yes	Yes	Yes	Yes	Yes	Yes	Yes	Low
Stagner et al. (68)	Yes	Yes	Yes	Yes	Yes	Yes	Yes	Yes	Low
Tallent et al. (69)	Yes	Yes	Unclear	Yes	Unclear	Yes	Yes	Yes	Low
Tarsitano et al. (70)	Yes	Unclear	Yes	Yes	Yes	Yes	No	Yes	High
Toferer et al. (71)	Yes	Yes	Yes	Yes	Yes	Yes	Yes	Yes	Low
Wendt et al. (72)	Yes	Yes	Yes	Yes	Yes	Yes	Yes	Yes	Low
Yazici et al. (73)	Yes	Yes	Yes	Yes	Yes	Yes	No	Yes	Low

Note: Q1: Were patient’s demographic characteristics clearly described? Q2. Was the patient’s history clearly described and presented as a timeline? Q3. Was the current clinical condition of the patient on presentation clearly described? Q4. Were diagnostic tests or assessment methods and the results clearly described? Q5. Was the intervention(s) or treatment procedure(s) clearly described? Q6. Was the post-intervention clinical condition clearly described? Q7. Were adverse events (harms) or unanticipated events identified and described? Q8. Does the case report provide takeaway lessons?

**Table 4:** Clinicopathological data of individuals with central giant cell granulomas of the jaws managed with pharmacological therapy retrieved in the present systematic review.

Variables	n (%)
Age
Mean ± SD (range)	18.1 ± 12.3 (0–80)
0-9 years	49 (23.9)
10-19 years	85 (41.5)
≥20 years	71 (34.6)
Sex
Female	125 (61.0)
Male	80 (39.0)
Swelling
Reported	74 (36.1)
Not reported	131 (63.9)
Pain
Reported	16 (7.8)
Not reported	189 (92.2)
Other signs/symptoms
Tooth mobility	10 (4.8)
Tooth displacement	8 (3.9)
Paresthesia	3 (1.5)
Cortical perforation	3 (1.4)
Evolution time
Reported	62 (21.7)
Not reported	143 (78.3)
Mean ± SD (median; range)	7.5 ± 8.6 (5.0; 0.2–48.0) months
Lesion behavior
Aggressive	122 (59.5)
Non-aggressive	83 (40.5)
Radiographic features
Radiolucent	96 (46.8)
Radiopaque	–
Mixed	7 (3.4)
Not reported	102 (49.8)
Anatomical location
Mandible	148 (72.2)
Maxilla	57 (27.8)
Topography
Anterior	91 (44.4)
Posterior	85 (41.5)
Anterior and posterior	1 (0.5)
Not reported	28 (13.6)
Previous surgical treatment
None	137 (66.8)
Curettage/enucleation	58 (28.3)
Recontouring	1 (0.5)
Resection	2 (1.0)
Not reported	7 (3.4)
Previous pharmacological therapy
None	196 (95.6)
Triamcinolone	7 (3.4)
Calcitonin	1 (0.5)
Not reported	1 (0.5)
Current pharmacological therapy
Triamcinolone	77 (37.5)
Interferon	50 (24.4)
Calcitonin	30 (14.7)
Denosumab	25 (12.2)
Combination of drugs	15 (7.3)
Other	8 (3.9)
Route of administration
Subcutaneous	93 (45.4)
Intralesional	80 (39.0)
Nasal	13 (6.3)
Intralesional + subcutaneous	11 (5.4)
Intralesional + nasal	5 (2.4)
Intralesional + nasal + oral	1 (0.5)
Subcutaneous + nasal	2 (1.0)
Adverse effects
Yes	20 (9.8)
No	185 (90.2)
Surgical procedure after pharmacological therapy
None	157 (76.6)
Curettage/enucleation	39 (19.0)
Resection	7 (3.4)
Not reported	2 (1.0)
Clinical/image resolution
Total	158 (77.1)
Partial	47 (22.9)
Follow-up period - mean ± SD, (median; range)	46.9 ± 34.8, (36.6; 3.6–201.6) months
Recurrence
Yes	14 (6.8)
No	190 (92.7)
Not reported	1 (0.5)
Follow-up period - mean ± SD, (median; range)	45.3 ± 34.7, (36.0; 3.0–201.6) months
Osteoplasty
Yes	16 (7.8)
No	189 (92.2)

Note: SD, standard deviation.

## Data Availability

The datasets used and/or analyzed during the current study are available from the corresponding author.
